# Epidural Analgesia During Labor and Neonatal Hypoxic-Ischemic Encephalopathy

**DOI:** 10.1001/jamanetworkopen.2024.33730

**Published:** 2024-09-16

**Authors:** Marie-Coralie Cornet, Michael W. Kuzniewicz, Aaron W. Scheffler, Stephanie L. Gaw, Peter Yeh, Thomas B. Newman, Yvonne W. Wu

**Affiliations:** 1 Department of Pediatrics, Benioff Children Hospital, University of California, San Francisco; 2 Department of Pediatrics, Kaiser Permanente Northern California, Oakland; 3 Division of Research, Kaiser Permanente Northern California, Oakland; 4 Department of Epidemiology & Biostatistics, University of California, San Francisco; 5 Department of Obstetrics, Gynecology & Reproductive Sciences, University of California, San Francisco; 6 Department of Anesthesiology, University of California, San Francisco; 7 Department of Neurology, University of California, San Francisco

## Abstract

**Importance:**

Epidural analgesia is used by approximately 70% of birthing persons in the US to alleviate labor pain and is a common cause of elevated temperature in the birthing parent during labor, which, in turn, is associated with adverse neonatal outcomes such as hypoxic-ischemic encephalopathy (HIE).

**Objective:**

To determine whether epidural analgesia is associated with increased risk of HIE after adjusting for the birthing person’s maximal temperature before epidural placement and for the propensity to get an epidural.

**Design, Setting, and Participants:**

This retrospective, population-based cohort study was conducted at 15 Kaiser Permanente Northern California hospitals. Participants included singleton neonates born at 35 weeks’ or later gestational age between 2012 and 2019. Elective cesarean deliveries and deliveries within 2 hours of hospital admission were excluded. Data analysis was performed from November 2022 to June 2024.

**Exposure:**

The primary exposure was epidural analgesia during labor.

**Main Outcomes and Measures:**

The primary outcome was HIE, defined as the presence of both neonatal acidosis (ie, pH <7 or base deficit ≥10) and encephalopathy. The presence and timing of epidural analgesia and demographic, pregnancy, and labor characteristics were extracted from electronic medical records. A propensity score for receiving epidural analgesia was created including demographic variables and comorbidities predating epidural placement. Logistic regression was used to evaluate the association between epidural analgesia and HIE, adjusting for maximal birthing parent’s temperature before epidural placement and the propensity for receiving an epidural.

**Results:**

Among 233 056 infants born at 35 weeks’ or later gestational age by vaginal or unplanned cesarean delivery after at least 2 hours of in-hospital labor, 177 603 (76%) were exposed to epidural analgesia and 439 (0.19%) had HIE. On unadjusted analysis, epidural analgesia was associated with an increased risk of maximal temperature greater than 38 °C during labor (risk ratio [RR], 8.58; 95% CI, 8.06-9.14). Each degree increase in maximal temperature during labor was associated with nearly triple the odds of HIE (odds ratio [OR], 2.82; 95% CI, 2.51-3.17). However, there was no significant association between epidural analgesia and the risk of HIE either on crude (RR, 1.21; 95% CI, 0.96-1.53) or adjusted (adjusted OR, 0.93; 95% CI, 0.73-1.17) analyses.

**Conclusions and Relevance:**

In this cohort study including more than 230 000 parent-infant dyads, epidural analgesia was associated with increased maximal temperature during labor, a known risk factor for HIE. However, epidural analgesia was not associated with increased odds of HIE.

## Key Points

**Question:**

Is epidural analgesia associated with an increased risk of hypoxic-ischemic encephalopathy in neonates?

**Findings:**

In this cohort study of 233 056 parent-infant dyads, birthing parents who received epidural analgesia had significantly higher maximal temperatures in labor. However, there was no significant association between epidural analgesia and hypoxic-ischemic encephalopathy in the offspring.

**Meaning:**

Although epidural analgesia was associated with fever in the birthing parent, it was not associated with hypoxic-ischemic encephalopathy in neonates.

## Teaser

This cohort study examines whether epidural analgesia is associated with increased risk of hypoxic-ischemic encephalopathy in neonates.

## Introduction

Epidural analgesia, the most effective technique for alleviating labor pain, is used by 60% to 73% of birthing persons in the US.^1,2^ Although epidural analgesia is generally considered safe, it has been associated with unwanted effects, such as hypotension, transient decreased uteroplacental blood flow, urinary retention, and increase in temperature in the birthing parent.^3^ Fever is present in 15% to 25% of patients who receive an epidural during labor, a 5 times higher rate than in untreated patients.^4,5^

Neonatal hypoxic-ischemic encephalopathy (HIE) is a neurologic syndrome caused by a lack of blood flow or oxygen to the brain around the time of birth, resulting in long-term neurodevelopmental impairment or death in one-half of the affected neonates.^6^ Parental fever during labor is thought to reduce the fetus’s ability to tolerate hypoxia-ischemia.^7^ In animal models, artificially increasing maternal temperature can cause fetal acidosis and neurotoxicity.^8,9^ Similarly, a small brain temperature increase in neonatal animals during or following hypoxia-ischemia exacerbates the extent of injury.^10,11^ Hyperthermia induces free radical production, glutamate and glycine release, and an increased metabolic demand, all of which can be deleterious to the neonatal brain.^12^ Parental fever and chorioamnionitis have been associated with an increased risk of HIE and cerebral palsy in a number of studies.^4,13,14,15^ Given that epidural analgesia is associated with increased parental temperature, it is possible that epidural analgesia increases the risk of HIE.^4^

Studies of epidural analgesia and adverse neonatal outcomes such as HIE are inconclusive. A 2018 Cochrane review^2^ found no difference in admission rates to intensive care nurseries or in Apgar scores between neonates exposed and unexposed to epidural analgesia, although the results were limited by imprecision in effect estimates and possible publication bias. Some studies^16,17^ suggest an association between epidural analgesia and lower Apgar scores and increased risk of birth injuries, fetal distress, and neonatal infection.^18,19^ However, because birthing persons with complex labor courses are more likely to receive epidural analgesia and to have adverse birth outcomes, there is a potential for confounding by indication.^2^ Available studies are further limited by small sample sizes,^4^ bias due to sample selection (eg, restricted to vaginal deliveries^20^ or nulliparous parents^16^), and the lack of detailed data on parental temperature and presence of HIE.^15,21^ In a large birth cohort, we examined the association between epidural analgesia and risk of HIE using detailed demographic, labor and delivery, and infant data to adjust for multiple potential confounders.

## Methods

### Study Population and Setting

This population-based cohort study included all singleton infants born at 35 weeks’ or later gestational age at 15 Kaiser Permanente Northern California (KPNC) hospitals between January 1, 2012, and July 31, 2019. We excluded 731 infants with congenital anomalies or genetic abnormalities, 35 390 infants born by elective cesarean delivery, and 20 506 infants of birthing parents who delivered within 2 hours of hospital admission because they would be unlikely to have received epidural analgesia.

KPNC is an integrated health care system serving more than 4.6 million members, representing approximately 40% of the insured population in Northern California. The sociodemographic distribution of the KPNC membership is broadly similar to that of the Northern California population, although the extremes of the income distribution are underrepresented.^22^ The University of California San Francisco and the Kaiser Foundation Research Institute institutional review boards approved this study and granted a waiver of individual consent, because the data are deidentified, in accordance with 45 CFR §46. Data were analyzed and reported in accordance with the Strengthening the Reporting of Observational Studies in Epidemiology (STROBE) reporting guidelines.^23^

### Exposures

The primary exposure of interest was epidural analgesia administered during labor. From the Kaiser Permanente Perinatal Research Unit database,^24,25^ we extracted timing of epidural placement and the following covariates: birth year; birthing parent’s self-reported race and ethnicity (ie, Asian, Black, Hispanic, White, and other, which includes American Indian, multiracial, and unknown); state-subsidized insurance; neighborhood deprivation index^26^ (a validated scale that measures aspects of poverty proven to be critical indicators of community health outcomes, with higher values indicating more deprivation); parental age, prepregnancy body mass index, parity, and complications (type 2 diabetes, gestational diabetes, chronic and gestational hypertension, anxiety or depression during pregnancy, preeclampsia, and a clinical diagnosis of chorioamnionitis); timing of rupture of membranes relative to delivery; birth hospital; neonatal intensive care unit acuity level (level 3 is highest, level 1 is lowest); and infant sex, birth weight, and gestational age. Data on race and ethnicity were included in this study because racial and ethnic disparities in access to epidural analgesia and neonatal outcomes could be important confounders in our analysis. Prolonged rupture of membranes was defined as 18 hours or longer before delivery. We collected all parental temperature measurements between 72 hours before delivery to 1 hour after delivery and defined the maximal parental temperature before epidural as follows: (1) for birthing parents who received an epidural, we used the maximal temperature before epidural placement; (2) for birthing parents who did not receive epidural analgesia, we used the maximal temperature during the first 7.5 hours after hospital admission, because the median (IQR) elapsed time from hospital admission to epidural placement was 7.5 (3.0-15.6) hours.

### Outcomes

Our primary outcome was HIE, defined as the presence of both neonatal acidosis and neonatal encephalopathy, as previously described.^27^ Briefly, neonatal acidosis was defined as at least 1 pH measurement less than 7 or base deficit greater than or equal to 10 from any cord blood, or a base deficit greater than or equal to 10 on the first infant blood gas before age 2 hours. Neonatal encephalopathy was confirmed by medical record review and was defined as an abnormal standardized neurologic examination^28^ between ages 1 and 6 hours that (1) persisted beyond age 6 hours, (2) was accompanied by seizures, or (3) was treated with active therapeutic hypothermia. Secondary outcomes included 5-minute Apgar score less than 7, neonatal acidosis, neonatal encephalopathy, treatment with therapeutic hypothermia, neonatal seizures, and neonatal culture-positive sepsis.

### Statistical Analysis

Data analysis was performed from November 2022 to June 2024. We estimated the odds ratio (OR) and 95% CI of epidural analgesia for risk of HIE using multivariable logistic regression and marginal absolute risk differences to capture population-level effects.^29^ Statistical significance was defined as a 95% CI that did not include the reference value of 1. To adjust for potential confounders, we included in our multivariable models (1) maximal parental temperature before epidural and (2) the propensity to receive an epidural as a spline.

We determined each birthing parent’s propensity to receive epidural analgesia by building a separate logistic regression model that included all potential confounding variables that were known before epidural analgesia placement (Figure): birth year, birth hospital, parental self-reported race and ethnicity, age, parity, state-subsidized insurance, neighborhood deprivation index, type 2 diabetes, gestational diabetes, chronic hypertension, preeclampsia, and anxiety or depression during pregnancy; and infant sex, birth weight, gestational age. The timing of ruptured membranes could occur either before or after initiation of epidural analgesia; thus, we divided the timing of ruptured membranes into 3 categories: (1) before hospital admission, (2) within 2 hours of admission, when rupture of membranes would unlikely occur after epidural placement, or (3) after 2 hours of hospitalization. We excluded from the multivariable analysis 535 infants (0.2%) who had missing data for covariates that were used to build the propensity score. All continuous variables were included as splines if they departed from linearity.

**Figure.**
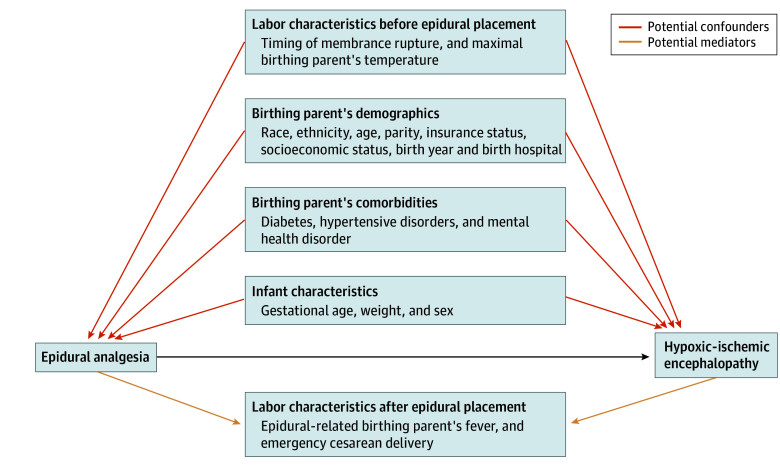
Directed Acyclic Graph of Potential Association Between Epidural Analgesia and Hypoxic-Ischemic Encephalopathy, Including Potential Confounders and Mediators The population included all infants born at 35 weeks’ gestation or later at 15 Kaiser Permanente Northern California hospitals and excluded infants born by elective cesarean delivery or within 2 hours of admission and those with congenital anomalies. To assess the potential causal pathway between epidural analgesia and hypoxic-ischemic encephalopathy, our multivariable model included potential confounders that were known before epidural placement and did not adjust for potential mediators.

Although birth weight is unknown during labor, it was included in the propensity score because it approximates estimated fetal weight, a variable that may influence the birthing parent’s choice to receive an epidural. In 3 separate sensitivity analyses, we (1) excluded birth weight from the propensity score, (2) adjusted for the duration of rupture of membranes and the maximal parental temperature up to epidural placement or 1 hour before delivery among parents who did not receive epidural analgesia, and (3) excluded all dyads where the birthing parent had a temperature greater than 37.5 °C before epidural analgesia placement. All analyses were clustered by hospital site using robust variance estimates from the clustered sandwich estimator. We checked that our models fit all underlying assumptions, including assessing covariate overlap, departure from linearity and spline adjustment as needed, and presence of interactions. Analyses were performed using Stata statistical software version 17 (StataCorp).

## Results

Our cohort study included 233 056 infants born at 35 weeks’ gestation or later between January 1, 2012, and July 31, 2019, among 15 hospitals in Northern California and their birthing parents. A total of 177 603 parents (76%) received epidural analgesia during labor. Parental characteristics associated with a higher frequency of epidural analgesia included Asian or White race, age younger than 35 years, nulliparity, coming from a neighborhood in the lowest deprivation index tertile (ie, less deprived neighborhood), and giving birth in a hospital with a level 3 neonatal intensive care unit (Table 1).

**Table 1.** Clinical Characteristics of 233 056 Parent-Infant Dyads Stratified by Use of Epidural Analgesia

Characteristic	Participants, No. (%)		RR (95% CI)^a^
	Total cohort (N = 233 056)	Received epidural analgesia (n = 177 603)	
Demographic characteristics			
Birthing parent’s self-reported race and ethnicity			
Asian	57 301 (24.6)	44 404 (77.5)	0.99 (0.98-1.01)
Black	14 925 (6.4)	11 298 (75.7)	0.97 (0.95-0.99)
Hispanic	60 785 (26.1)	44 130 (72.6)	0.93 (0.92-0.94)
White	86 740 (37.2)	67 640 (78.0)	1 [Reference]
Other^b^	13 305 (5.7)	10 131 (76.1)	0.98 (0.98-1.00)
Birthing parent’s age			
<35 y	189 540 (78.7)	145 915 (77.0)	1 [Reference]
≥35 y	43 516 (18.7)	31 688 (72.8)	0.95 (0.93-0.96)
Parity			
Nulliparous	111 498 (47.8)	90 896 (81.5)	1 [Reference]
1	77 532 (33.3)	57 844 (74.6)	0.92 (0.91-0.92)
2	29 126 (12.5)	19 442 (66.8)	0.82 (0.81-0.83)
≥3	14 900 (6.4)	9421 (63.2)	0.78 (0.76-0.79)
State subsidized insurance	23 399 (10.0)	17 513 (74.8)	0.98 (0.97-1.00)
Neighborhood deprivation index			
First tertile (least deprived)	77 194 (33.1)	60 891 (78.9)	1.03 (1.02-1.04)
Second tertile	77 406 (33.2)	59 461 (76.8)	1 [Reference]
Third tertile (most deprived)	78 456 (33.7)	57 251 (73.0)	0.95 (0.94-0.96)
Neonatal intensive care unit facility level of care			
Level 1	5651 (2.4)	3941 (69.7)	0.89 (0.86-0.92)
Level 2	83 379 (35.8)	60 627 (72.7)	0.93 (0.92-0.94)
Level 3	144 026 (61.8)	113 035 (78.5)	1 [Reference]
Pregnancy comorbidities			
Type 2 diabetes	2381 (1.0)	1632 (68.5)	0.90 (0.86-0.94)
Gestational diabetes	26 240 (11.3)	19 663 (74.9)	0.98 (0.97-1.00)
Chronic hypertension	9363 (4.0)	6927 (74.0)	0.97 (0.95-0.99)
Preeclampsia	14 171 (6.1)	10 400 (73.4)	0.96 (0.94-0.98)
Anxiety diagnosis during pregnancy	21 672 (9.3)	17 516 (80.8)	1.07 (1.05-1.08)
Depression diagnosis during pregnancy	36 037 (15.5)	28 828 (80.0)	1.06 (1.05-1.07)
In-utero growth restriction	6113 (2.6)	4299 (70.3)	0.92 (0.89-0.95)
Labor characteristics			
Maximal parental temperature before epidural placement^c^			
<37.0 °C	121 277 (52.0)	90 119 (74.3)	1 [Reference]
37-37.5 °C	97 175 (41.7)	74 721 (76.9)	1.03 (1.02-1.04)
37.5-38 °C	10 551 (4.5)	9007 (85.4)	1.15 (1.12-1.17)
38-38.5 °C	2738 (1.2)	2545 (93.0)	1.25 (1.20-1.30)
38.5-39 °C	959 (0.4)	902 (94.1)	1.26 (1.19-1.35)
>39 °C	356 (0.2)	309 (86.8)	1.17 (1.04-1.31)
Rupture of membranes			
Before hospital admission	64 008 (27.5)	48 837 (76.3)	0.99 (0.98-1.00)
Within 2 h of admission	11 697 (5.0)	7481 (64.0)	0.83 (0.81-0.85)
After 2 h of admission	157 351 (67.5)	121 285 (77.1)	1 [Reference]
Infant characteristics			
Sex			
Female	113 260 (49.6)	85 930 (75.9)	0.99 (0.98-1.00)
Male	119 796 (51.4)	91 673 (76.5)	1 [Reference]
Gestational age			
Late preterm (35-36 wk)	9539 (4.1)	6145 (64.4)	0.85 (0.83-0.87)
Full term (37-40 wk)	190 610 (81.8)	144 593 (75.9)	1 [Reference]
Postterm (41-44 wk)	32 907 (14.1)	26 865 (81.6)	1.08 (1.06-1.09)
Birth weight			
<3000 g	45 150 (19.4)	32 441 (71.9)	0.93 (0.92-0.94)
3000-4000 g	164 162 (70.4)	126 439 (77.0)	1 [Reference]
>4000 g	23 744 (10.2)	18 722 (78.8)	1.02 (1.01-1.04)

Abbreviation: RR, risk ratio.

^a^ For categorical variables, the reference group is the group with the most individuals.

^b^ Other includes American Indian, multiracial, and unknown.

^c^ For birthing parents who did not receive epidural analgesia, this refers to maximal temperature during the first 7.5 hours after hospital admission.

During labor, parents who received epidural analgesia had higher rates of temperature above 38 °C (risk ratio [RR], 8.58; 95% CI, 8.06-9.14) and higher mean maximal temperatures and were at higher risk of receiving a clinical diagnosis of chorioamnionitis (Table 2). Parents who received epidural analgesia also had a longer mean elapsed time between hospital admission and time of delivery and a higher rate of prolonged rupture of membranes (Table 2).

**Table 2.** Parental Temperature, Chorioamnionitis, and Duration of Labor Stratified by Presence of Epidural Analgesia

Variable	Patients, No. (%)		Result (95% CI)
	Epidural analgesia (n = 177 603)	No epidural analgesia (n = 55 453)	
Birthing parent temperature			
Maximal temperature in labor, mean (SD), °C	37.40 (0.56)	37.00 (0.33)	0.35 (0.35-0.36)^a^
Maximal temperature minus admission temperature, mean (SD), °C	0.66 (0.59)	0.30 (0.35)	0.36 (0.35-0.36)^a^
Maximal temperature ≥38.0 °C	26 905 (15.1)	979 (1.8)	8.58 (8.06-9.14)^b^
Clinical diagnosis of chorioamnionitis	23 176 (13.0)	941 (1.7)	7.70 (7.20-8.20)^b^
Progression of labor			
Elapsed time from hospital admission to delivery, median (IQR), h	14.7 (9.0-23.4)	7.2 (3.9-13.0)	7.5 (7.4-7.6)^c^
Elapsed time from hospital admission to delivery >24 h	42 366 (23.9)	4588 (8.3)	2.90 (2.80-3.00)^b^
Prolonged rupture of membranes (>18 h)	36 704 (20.7)	6728 (12.1)	1.70 (1.70-1.70)^b^

Abbreviation: RR, risk ratio.

^a^ Data are mean difference.

^b^ Data are RR.

^c^ Data are median difference.

We identified 3892 infants (16.7 per 1000) with neonatal acidosis and 530 infants (2.27 per 1000) with neonatal encephalopathy. A total of 439 infants (0.19%) who had both neonatal acidosis and neonatal encephalopathy met our study criteria for HIE (population incidence 1.88 per 1000).

The incidence of HIE was not significantly different between infants exposed (1.97 per 1000) and unexposed (1.62 per 1000) to epidural analgesia (RR, 1.21; 95% CI, 0.96-1.53). However, elevated parental temperature during labor was associated with HIE. Specifically, each degree increase in maximal parental temperature during labor was associated with a nearly 3-fold increase in the odds of neonatal HIE (OR, 2.82; 95% CI, 2.51-3.17).

After adjusting for the propensity for receiving an epidural and maximal parental temperature before epidural placement, we did not find a significant association between epidural analgesia and HIE (adjusted OR [aOR], 0.93; 95% CI, 0.73-1.17). In sensitivity analyses, there remained no significant association between epidural analgesia and HIE after (1) removing birth weight from the propensity model (aOR, 0.92; 95% CI, 0.73-1.16), (2) including all variables up to the time of epidural placement, or up to 1 hour before delivery in birthing parents without epidural analgesia (aOR, 0.95; 95% CI, 0.73-1.23), or (3) excluding all birthing parents with a temperature greater than 37.5 °C before epidural placement (aOR, 0.87; 95% CI, 0.69-1.11).

In secondary crude analyses, epidural analgesia was associated with an increased risk of 5-minute Apgar score less than 7 and an increased risk of neonatal encephalopathy (Table 3). After adjusting for the propensity for receiving an epidural, epidural analgesia was associated with having a 5-minute Apgar score less than 7 (aOR, 1.38; 95% CI, 1.27-1.49), but not with other secondary outcomes.

**Table 3.** Associations Between Epidural Analgesia and Hypoxic-Ischemic Encephalopathy and Secondary Outcomes

Outcome	Neonates, No. (%)		Unadjusted RR (95% CI)	Adjusted OR (95% CI)^a^	Adjusted RD/1000 births (95% CI)^b^
	Epidural analgesia (n = 177 603)	No epidural analgesia (n = 55 453)			
Hypoxic-ischemic encephalopathy	349 (0.20)	90 (0.16)	1.21 (0.96 to 1.53)	0.93 (0.73 to 1.17)	−0.2 (−0.6 to 0.3)
Apgar score <7 at 5 min	2273 (1.28)	429 (0.77)	1.65 (1.49 to 1.83)	1.38 (1.27 to 1.50)	3.4 (2.5 to 4.3)
Neonatal acidosis^c^	2992 (1.68)	900 (1.62)	1.04 (0.96 to 1.12)	0.85 (0.73 to 0.99)	−2.8 (−5.2 to −0.3)
Neonatal encephalopathy	426 (0.24)	107 (0.19)	1.24 (1.01 to 1.54)	0.97 (0.80 to 1.17)	−0.1 (−0.5 to 0.4)
Therapeutic hypothermia	273 (0.15)	76 (0.14)	1.12 (0.87 to 1.45)	0.83 (0.68 to 1.02)	−0.3 (−0.6 to 0.1)
Neonatal seizures	171 (0.10)	53 (0.10)	1.00 (0.74 to 1.37)	0.81 (0.62 to 1.05)	−0.2 (−0.5 to 0.1)
Neonatal culture positive sepsis	54 (0.03)	15 (0.03)	1.13 (0.64 to 2.00)	1.06 (0.59 to 1.91)	0.0 (−0.2 to 0.2)

Abbreviations: OR, odds ratio; RD, risk difference; RR, risk ratio.

^a^ ORs are adjusted for the propensity for receiving an epidural and the maximal maternal temperature before epidural placement. Given the low prevalence of outcomes, the ORs approximate the RRs.

^b^ Adjusted RDs are calculated by estimating the probability of hypoxic-ischemic encephalopathy for each participant with or without epidural analgesia according to their observed propensity for receiving an epidural and maximal parental temperature before epidural placement, then taking the difference of the estimated probabilities, and averaging these RDs across the sample.

^c^ Neonatal acidosis is defined as cord blood pH less than 7 or base deficit greater than or equal to 10, or base deficit greater than or equal to 10 on first infant gas.

## Discussion

In a large population-based birth cohort study, we found that epidural analgesia was associated with increased parental temperature during labor. Furthermore, increased parental temperature was associated with a higher risk of HIE. However, we did not find an association between epidural analgesia and the risk of HIE.

Labor pain is described as one of the most painful experiences in a birthing individual’s life. Epidural analgesia is the most effective treatment for labor pain, and its positive impact on parental experiences of labor is well recognized.^3^ We confirm previous findings^2,4,30^ that epidural analgesia is associated with increased parental temperatures and prolonged labor. Epidural-related parental fever is caused by both the epidural itself and other confounding factors, such as labor duration, labor dystocia, and other parental comorbidities associated with the choice to request epidural analgesia. Thus, it is important to adjust for the propensity to receive an epidural when evaluating the association between epidural analgesia and neonatal outcomes.

We found that for each degree increase in maximal parental temperature during labor, there was a nearly 3-fold increase in the adjusted odds of HIE. Previous studies^4,14,31^ have similarly found an association between parental fever during labor or chorioamnionitis and adverse neurological outcomes in neonates, such as HIE and neonatal brain injury. However, even though epidural analgesia was associated with higher parental temperatures, we did not find a significant association between epidural analgesia and HIE. There are several potential reasons for this negative finding. First, the elevated parental temperatures resulting from epidural analgesia may be less deleterious to the fetus than parental fevers resulting from other causes, such as infection. Epidural-related parental fever is specific to birthing persons and may be mediated by a noninfectious inflammatory process and thermal dysregulation.^5^ Second, epidural analgesia has been shown to facilitate second-stage assisted deliveries by achieving a surgical anesthetic level in minutes, thus often avoiding delays or the need for general anesthesia in the setting of fetal distress.^32^ Third, there is some evidence that epidural analgesia may promote improved placental perfusion,^33^ which could reduce the degree of hypoxia-ischemia to the fetus. Finally, there may be other mechanisms by which epidural is neuroprotective during labor.

A key challenge in assessing the association between epidural analgesia and HIE is confounding by indication.^16,34^ We found that birthing parents exposed to epidural analgesia had longer labor, more frequent diagnoses of chorioamnionitis, and longer duration of ruptured membranes (Table 2). One of our study’s strengths is the ability to adjust for indicators of labor dystocia, such as the timing of membrane rupture and maximal parental temperature before epidural placement. Note that because epidural analgesia increases parental temperature, maximal parental temperature at any time during labor is a potential mediator of the association between epidural and HIE (Figure). A previous study^16^ found that epidural analgesia was associated with neonatal encephalopathy on crude analysis but not after adjusting for parental fever at any time during labor; it is possible, however, that by adjusting for this potential mediator, the study negated a true association. Similarly, because obstetricians diagnose chorioamnionitis on the basis of clinical signs such as fever, adjusting for, or stratifying by chorioamnionitis, could bias the analysis. In this study, which did not adjust for such mediators, we confirmed that there is no significant association between epidural analgesia and HIE. Although many cases of HIE remain unexplained, giving rise to frequent medicolegal debate,^35^ our findings suggest that epidural analgesia is not a risk factor for HIE.

Another strength of our study is that we excluded birthing parents who could not feasibly receive epidural analgesia during labor. In contrast to studies^4,16,17,34^ that rely on administrative datasets, we were able to exclude parents who delivered so quickly after hospital admission (ie, within 2 hours) that they were unlikely to have had time to receive epidural analgesia. Similarly, we excluded birthing parents who delivered via elective cesarean delivery because they were both unlikely to receive epidural analgesia and unlikely to have an infant with HIE. Including these parents would introduce a bias toward the null. Finally, our analyses included both vaginal and cesarean deliveries after labor, because excluding these cesarean deliveries could lead to bias (Figure).^20^

In secondary analyses, we found that epidural analgesia was associated with a higher risk of low 5-minute Apgar score. Other studies^16,17^ have reported a similar association between epidural analgesia and low Apgar scores. However, even if epidural analgesia were causally related to a low 5-minute Apgar score, the adjusted risk difference is very low (Table 3) and, hence, the potential number needed to harm would be high. Furthermore, a low 5-minute Apgar score is most often a transient finding, with expected full recovery after resuscitation in most cases.^36^ Of note, our secondary exploratory analyses did not account for multiple comparisons, and it is possible that the higher risk of a low 5-minute Apgar score is a chance finding. Interestingly, despite a higher rate of chorioamnionitis diagnoses among parents who received epidural analgesia, there was no increased risk of neonatal culture-proven sepsis. The increased rate of chorioamnionitis in parents exposed to epidural analgesia is likely due to epidural-related parental fever and confounding by indication, as there is no reason to believe that epidural analgesia causes chorioamnionitis.

### Limitations

Our study has several limitations. Like all observational studies, our analyses may be limited by unmeasured confounders. However, given the low incidence of HIE, a randomized study would require a very large sample size. Furthermore, it is challenging to randomize birthing parents to receive an epidural or not. Hence, the only way to assess potential harm from epidural analgesia is with observational studies, carefully considering potential confounders and mediators.^37^ Second, our propensity model is limited, because we lacked information on important factors such as parental choice regarding epidural analgesia and clinician practice preferences. We also lacked information regarding the progression of labor before epidural placement. In addition, although we did not find a significant association between epidural analgesia and risk of HIE, the 95% CI of the aOR extends from 0.73 to 1.17. As noted in Table 3, even if there were a statistically significant causal association between epidural analgesia and HIE within this range of effect sizes, the absolute risk differences would be minimal and not clinically meaningful.

## Conclusions

Epidural analgesia is the criterion standard for pain management in labor and improves a birthing parent’s experience. In a large birth cohort, we confirmed that epidural analgesia was associated with increased maximal parental temperature during labor, but we did not find a significant association between epidural analgesia and HIE, suggesting that epidural analgesia is not a risk factor for HIE.

## Notes

Supplement.
Data Sharing Statement
