# Supplementary material for: Epidural Analgesia During Labor and Neonatal Hypoxic-Ischemic Encephalopathy
Source: JAMA Netw Open. 2024 Sep 16;7(9):e2433730. doi: 10.1001/jamanetworkopen.2024.33730 (PMC11406397; doi:10.1001/jamanetworkopen.2024.33730)
Supplement: Supplement. — Data Sharing Statement [file jamanetwopen-e2433730-s001.pdf]

Data Sharing Statement

Cornet. Epidural Analgesia During Labor and Neonatal Hypoxic-Ischemic Encephalopathy. *JAMA Netw Open*. Published September 16, 2024. doi:10.1001/jamanetworkopen.2024.33730

Data
Data available: No

Additional Information
Explanation for why data not available: Data are available upon reasonable request. The datasets generated for this study are stored at the KPNC Division of Research. Deidentified data can be provided upon reasonable request to the corresponding author, and with permission from the KPNC Institutional Review Board.
